# 

**Published:** 1891-04

**Authors:** 


CONTENTS:

PAGE

ORIGINAL ARTICLES:

Meningitis. By J. C. Michener, M. D., V. S. .......... 157
Tracheotomy and Laryngeal Injections in the Throat. By J. C. Meyers, M. D., V. S.......... 164
El Dourine. By W. H. Ridge, V. M. D. .......... 171
Age of the Horse, Ox, Dog, and Other Domesticated Animals. By R. S. Huidekoper, M. D., Veterinarian.......... 173
Recent Patents. .......... 171
Pseudo Tuberculosis. By Dr. A. W. Clement..........

EDITORIAL:

Reviews. .......... 187
Koch's Lymph in Bovine Tuberculosis .......... 188

SELECTIONS FROM FOREIGN JOURNALS:

German Review.......... 189

SOCIETY PROCEEDINGS:

Illinois State Veterinary Medical Association.......... 191
Pennsylvania State Veterinary Medical Association.......... 195
Nebraska Veterinary Medical Association. .......... 201
Massachusettes Veterinary Association.......... 201
Maryland State Veterinary Medical Society.......... 202
Society of Veterinary Graduates of Wisconsin.......... 203
Alumni Association, American Veterinary College.......... 203

VETERINARY COLLEGES:

American Veterinary College Commencement.......... 204
Chicago Veterinary College Commencement.......... 206

REVIEW DEPARTMENT:

Books and Pamphlets Received.......... 207

